# Prevalence of *Trichomonas vaginalis* Infection in Hamadan City, Western Iran

**Published:** 2012

**Authors:** M Matini, S Rezaie, M Mohebali, AH Maghsood, S Rabiee, M Fallah, M Rezaeian

**Affiliations:** 1 Dept. of Medical Parasitology and Mycology, School of Public Health, Tehran University of Medical Sciences, Tehran, Iran; 2 Center for Research of Endemic Parasites of Iran (CREPI), Tehran University of Medical Sciences, Tehran, Iran; 3 Dept. of Medical Parasitology and Mycology, School of Medicine, Hamadan University of Medical Sciences, Hamadan, Iran; 4 Dept. of Obstetrics and Gynecology, Fatemieh Women Hospital, School of Medicine, Hamadan University of Medical Sciences, Hamadan, Iran

**Keywords:** *Trichomonasvaginalis* infection, Wet mount, Culture, Iran

## Abstract

**Background:**

Infection with *Trichomonas vaginalis* is one of the most common sexually transmitted diseases (STDs) in humans. The prevalence of infection in Iran has been reported between 2 to 8%, depending on deferent socio-cultural conditions. This study aimed to determine the prevalence of *T*. *vaginalis* in women referred to gynecologic clinics in Hamadan city, West of Iran.

**Methods:**

This descriptive cross-sectional study was conducted on 750 women who referred to Gynecologic clinics in Hamadan from November 2010 to July 2011. Vaginal samples were obtained from them and examined by wet mount and culture methods for the detection of *T. vaginalis*.

**Results:**

Sixteen out of 750 vaginal swab specimens (2.1%) were culture positive for *T. vaginalis* and 13 of these positive specimens (1.7%) were wet mount positive. Only 12 of 42 patients who were clinically diagnosed as having *T. vaginalis* infection, confirmed by culture method. Five hundred and fifty of the participants women (73.3%) had at least one of signs and symptoms of trichomoniasis. No statistical correlation was observed between clinical manifestations and parasitological results (*p*>0.05).

**Conclusion:**

This study showed low prevalence of *T. vaginalis* infection in the study population. Since clinical signs of trichomonal vaginitis are the same of other STDs, a confirmatory laboratory diagnosis is necessary. Wet smear as well as culture are sensitive for detection of *T. vaginalis*.

## Introduction

*Trichomonas vaginalis* is a flagellated protozoan that causes trichomoniasis in human, a sexually transmitted disease (STD) with worldwide (1). Trichomoniasis is usually concomitant with other STDs, particularly gonorrhae, and indicates high-risk sexual manner (2). Presumably, prevalence of infection with *T. vaginalis* in nonselected population of women is 5%-20% and it is related to the target population so that the highest estimates are referred to those attending the sexual disease clinics (3). In 2001, World Health Organization (WHO) has estimated more than 170 million people to be infected annually with *T. vaginalis* throughout the world (4).

In the United States, prevalence of *T. vaginalis* among sexually active women was appraised 2 to 3 million symptomatic infection annually (5). In Iran, various studies have determined the prevalence of trichomoniasis between 2% to 8% that according to the cultural and social status can also reach over 30% (6). Trichomoniasis is associated clinically with preterm birth, low birth weight, infertility, pelvic inflammatory disease (1, 7, 8) hepatitis viruses, *Mycoplasma hominis* (8), incidence of cervical cancer and increased risk of Human Immunodeficiency Virus (HIV) transmission (5, 7, 9, 10). Clinical features of trichomoniasis in women vary and it may be seen from the symptomatic to asymptomatic forms (11). The prevalence of trichomoniasis and clinical features in men are poorly understood and its complications can be pointed as prostatitis, balanoposthitis, epididymitis and infertility (11, 12).

Previous studies in Hamadan City have been conducted only on symptomatic women, but the main objective of this study was to determine the prevalence of *T*. *vaginalis* infection in both the symptomatic and the asymptomatic women.

## Materials and Methods

This descriptive cross-sectional study was carried out on 750 of women in Fatemieh Hospital, Hamada and 9 government and private clinics in Hamadan City, west of Iran, from November 2010 to July 2011. All of the women came to the obstetrics and gynecologic clinics for receiving health care services or treatment of disease.

The referred women who had used vaginal agents and consumption of antibiotic during the past two weeks were excluded from the study. After obtaining informed consent from all referred individuals, demographic information such as age, education, occupation, husband's occupation, pregnancy, and clinical signs and symptoms including vaginal discharge, the color and consistency of discharge, itching, dysuria, dyspareunia, and inflammation of the genital tract were collected through interview and clinical examination by the gynecologist or midwife and the data were recorded in the questionnaires. Diagnostic methods were wet mount and culture technique, which the latter method was considered as the gold standard method (2, 11). Sampling was performed by two sterile cotton tipped swabs from vagina wall and dorsal fornix. One sample swab was put in a tube containing 0.5 ml of Ringer serum and the other was placed in liquid phase of Dorset medium (13). The sample tubes containing Ringer serum were transferred immediately to the laboratory in the clinics and subjected to wet mount examination using light microscope with low (100X) and high (400X) magnifications. Dorset culture medium was sent as soon as possible to the specialized laboratory of parasitology, Hamadan University of Medical Sciences and was incubated at 37 °C. After 24 h, a drop of sediment of the culture medium was removed and examined by direct smear. The culture medium was tested daily up to 7 days until they turned positive (14).

Chi-squared (χ^2^) and Fisher exact tests were used to compare *T. vaginalis* infection relative to clinical manifestations and parasitological results. Analyses were performed with SPSS (version 13.5; SPSS Inc, Chicago,IL, USA) and Epi-Info software, with a probability (*P*) value of <0.05 were considered as statistically significant.

## Results

*T. vaginalis* was detected in 16 out of 750 participants (2.1%) (95% CI, 1.1%-3.1%) by using culture methods whereas only 13 of 16 infected people were positive with the wet mount technique (1.7%) (Table 1).

**Table 1:** Detection of *Trichomonas vaginalis* in vaginal secretion by parasitological method

Method	Positive n (%)	Negative n (%)	Total
**Culture**	16 (2.1)	734 (97.9)	750
**Wet mount**	13 ( 1 .7)	737 (98.3)	750

Infected individual's age ranged from 25 to 46 years old with a mean of 34. The highest infection rate was in the age group 25-34 years (10/16, 62.5%) that was statistically significant (*P*<0.05). Fifteen of those infected were married housewives and one was widowed. More of the infected individuals with *T. vaginalis* (8 /16, 50%) had primary school education (*P*<0.05).

Forty-two women infected with *T. vaginalis* were detected by clinical examination whereas 12 of them were confirmed with culture and wet mount method. Four asymptomatic infected women were not identified by clinical examination. The sensitivity, specificity and positive and negative predictive value of clinical diagnosis compared to culture method were 75%, 95%, 28% and 99%, respectively. There was no statistical correlation between clinical and parasitological diagnosis methods.

**Fig. 1:**
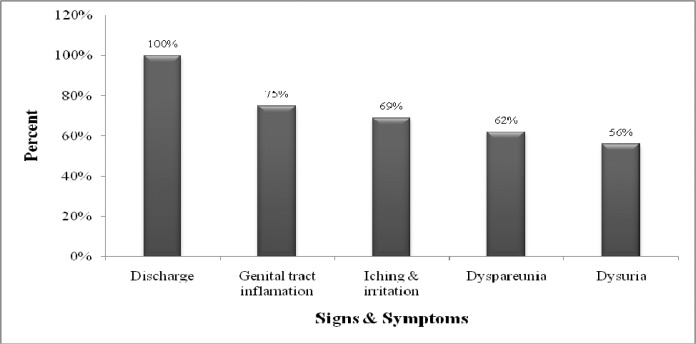
The predominant clinical signs and symptoms of patients infected with *Trichomonas vaginalis*

Candidiasis was observed in 113 women (15%), based on microscopic method using 10% Potassium hydroxide and gram stained smear. Five hundred and fifty (73.3%) of participants showed one or more signs and symptoms of trichomoniasis. The most common sign was vaginal discharge (71.3%). These signs and symptoms are not only specific for trichomoniasis, but also are common in all sexual transmitted disease. The most predominant signs and symptoms included vaginal discharge, itching, dysuria and inflammation of the genital tract, were observed (Fig. 1). No statistically correlation was observed between culture and wet mount for the detection of *T.vaginalis* infection.

## Discussion

Trichomoniasis is the most common non-viral sexual infection that causes infection in genitourinary tract (15, 16). Infection is mainly transmitted through sexual activity, although non-sexual transmission is also reported (6). Almost 2%-17% of female infants may be infected by their mothers (11). In the United States, the prevalence of trichomoniasis has been estimated at 25% in those referred to STDs clinics and it is higher in certain population groups such as African American women (38%) (2). Some studies have mentioned a 25% or higher infection rate in Africa (2). For instance, the prevalence of trichomoniasis in HIV-positive women in Zaire and pregnant women in a rural area in South Africa is estimated 38% and 65%, respectively (1, 2). In Islamic countries, the prevalence of trichomoniasis ranges from 1.2% in Libya and Jordan, 3.2% in Turkey to 28.1% in Saudi Arabia (17–20).

Like other parts of the world, the reports of trichomoniasis in Iran are different. For example, the prevalence of trichomoniasis in some areas of Iran include: 9.2% in Tabriz (21), 3.2% in Tehran (22), 4% in Babol (23), 2% in Yazd (24), 5.6% in Mashhad, 1.37% in Charmahal Bakhtiari province, 17% in Zahedan and 10.7% in Bandar Abbas (25). In this study, prevalence of trichomoniasis was 2.1% by using the culture method as the gold standard. The result of this study is similar to two other studies conducted in Hamadan in 2005 (3%) (26) and 2007 (2.2%) (25), but is different from the study in 2006 (18.1%) (27). Difference in results may be due to the selection of different population groups. Therefore, it is necessary to pay attention to special population sub-groups in certain communities, because of the wide variety in prevalence rates of trichomoniasis in the world. Diagnosis of trichomoniasis based on only clinical symptoms should not be done due to the two reasons. First, clinical symptoms of trichomoniasis may be similar to those of other STDs. Second, clinical symptoms such as strawberry cervix and spumy discharge are seen in 2% and 12% of *T. vaginalis* infected patients, respectively (11). Some studies have indicated that diagnosis based solely on clinical examination show 88% false negative and 29% false positive (11). As the most important point of this study, 73.3% of participants had clinical signs and symptoms. Forty-two of them were clinically diagnosed infected with *T. vaginalis*, in which only 12 were confirmed by culture technique. Also 4 of the infected patients were asymptomatic, which were not diagnosed by clinical examination. In this study, positive predictive value for clinical diagnosis of *T. vaginalis* infection was 28%, so the use of the laboratory diagnosis methods is necessary. The most common method for diagnosis of *T. vaginalis* is wet mount, but its sensitivity has been reported between 38% and 82%. Molecular method based on PCR is an accurate method with a sensitivity of 80% to 100%, but it is not routine in all laboratories (2, 11, 28).

In conclusion, the results of this study shows the prevalence of *T. vaginalis* infection in the study population is relatively low and other causes of vaginitis such as bacterial and fungal infections should be more consideration. Since clinical signs of trichomonal vaginitis are the same of other STDs, a confirmatory laboratory diagnosis is necessary. Further studies in different population groups are needed to determine other aspects of epidemiology of this infection in Iran.
